# Supplementary material for: 4-Methylumebelliferone Enhances Radiosensitizing Effects of Radioresistant Oral Squamous Cell Carcinoma Cells via Hyaluronan Synthase 3 Suppression
Source: Cells. 2022 Nov 25;11(23):3780. doi: 10.3390/cells11233780 (PMC9741296; doi:10.3390/cells11233780)
Supplement: Supplementary file 1 [file cells-11-03780-s001.zip › cells-2005014-supplementary.pdf]

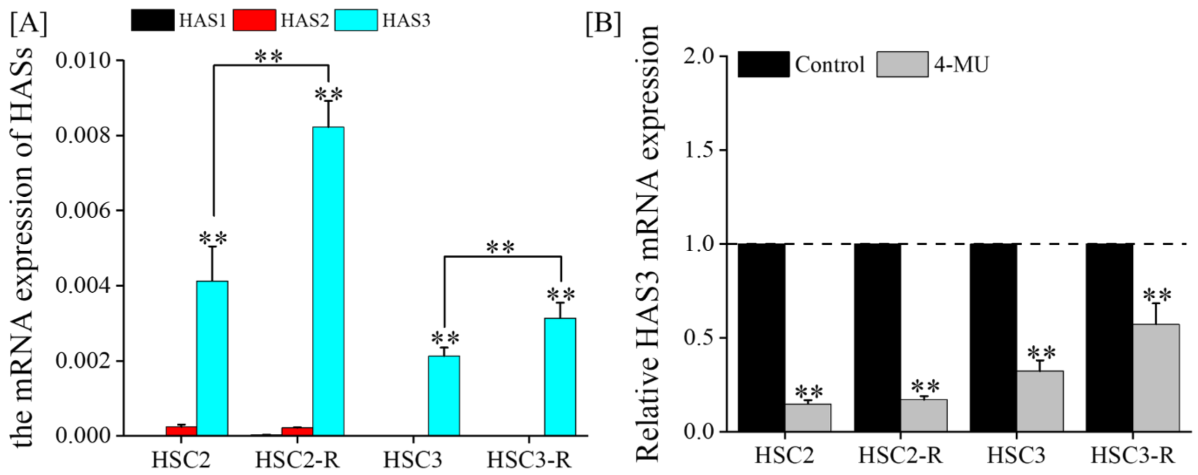

Figure S1. The mRNA expression of HAS as measured by RT-qPCR. A) mRNA expression of HAS in each cell line. (B) Relative mRNA expression of HAS3 treated with 500 μM 4-MU. ** indicates P < 0.01 vs. control.
